# Psychosocial health of asylum seeking women living in state-provided accommodation in Germany during pregnancy and early motherhood: A case study exploring the role of social determinants of health

**DOI:** 10.1371/journal.pone.0208007

**Published:** 2018-12-28

**Authors:** Sandra Claudia Gewalt, Sarah Berger, Sandra Ziegler, Joachim Szecsenyi, Kayvan Bozorgmehr

**Affiliations:** Department of General Practice & Health Services Research, University Hospital Heidelberg, Heidelberg, Germany; Medizinische Universitat Wien, AUSTRIA

## Abstract

**Background:**

Pregnant women and new mothers seeking asylum are subject to major challenges that may affect their health and increase their vulnerability. The study aim was to investigate asylum seeking women’s experiences and perceived needs during pregnancy and early motherhood whilst living in state-provided accommodation in one federal state in Southern Germany, with a particular focus on psychosocial factors.

**Methods:**

In this exploratory case study, individual semi-structured interviews were conducted with participants in pregnancy and up to the six-week postnatal assessment. Two female interviewers performed interviews assisted by female professional interpreters. Interviews were recorded digitally and transcribed verbatim. An inductive approach was taken to content analysis of interview material.

**Results:**

21 interviews were performed with nine women seeking asylum in pregnancy and early motherhood. Women shared experiences and perceived consequences on their psychosocial health. The following five key themes were identified during content analysis: a) psychosocial stressors, b) stressful living circumstances, c) stressful relationships, d) social support and e) coping styles. Psychosocial factors were a significant source of mental stress for participants, especially due to future uncertainties linked with the asylum seeking process. Living circumstances were also marked by stressors including a lack of privacy, verbal and physical threats and experiences of powerlessness. Further strain and emotional pressure were caused by stressful relationships with the unborn child’s father. Social support and personal coping styles provided relief for some participants.

**Conclusions:**

This study provides in-depth insights into the experiences and perceived needs of pregnant asylum seekers and new mothers living in state-provided accommodation. Key results identified psychosocial factors such as future uncertainties, stressful living circumstances and stressful relationships, as social determinants of health that were perceived to adversely affect women’s health. Adequate social support and individual coping styles increased resilience and counterbalanced psychosocial stressors during the asylum seeking process.

## Background

Germany has experienced a significant increase in the number of asylum seekers in recent years, with a total of 745,545 persons applying for asylum in 2016 [1]. An asylum seeker is an individual who has sought international protection and whose claim for refugee status has not yet been determined [2]. The experiences of refugees and asylum seekers during the migrant trajectory cause significant psychosocial stress. Psychosocial stress has been defined as an imbalance between requests placed on us and our aptitude to manage them [3, 4]. This feeling of being unable to cope with demands fuels the experience of stress, expressed behaviorally, physiologically and psychosocially. Psychosocial stress places a burden on mental health, which is understood as “a state of well-being in which every individual realizes his or her own potential and can cope with the normal stresses of life” [5]. During the migrant trajectory, refugees and asylum seekers experience multiple stressors over and above everyday issues, which impose major challenges on their health.

Asylum seeking women in particular are “highly vulnerable and socially excluded”, as Asif, Baugh and Jones (2015) highlight [6], especially during pregnancy and early motherhood. Psychosocial stress, experienced by pregnant women, was associated with poor pregnancy outcomes [7]. Examples of psychosocial stressors that can negatively impact on women in the prenatal period include: depression, panic disorder, substance abuse, domestic violence and having two or more medical comorbidities [7]. Such psychosocial stressors are compounded for pregnant women seeking asylum. Studies showed that asylum seeking women who are pregnant or in their postnatal period (including the first six weeks after delivery), also referred to as early motherhood, suffer from an increased risk of severe complications [8], an increased risk of poor maternal health outcomes [9], an elevated risk of severe maternal morbidity and an increased risk of perinatal and maternal mortality [10]. Compared to the general population, maternal mortality in asylum seekers and refugees is three times and postnatal depression is four times more likely [6]. Looking at the overall population of migrants, results from systematic reviews found that one in three women suffered from depressive disorders during pregnancy and early motherhood [11] and that these women were considered particularly vulnerable to suffer from depression when facing social isolation and poor language proficiency of the host country [12]. Psychosocial stressors impact on both physical and mental health and are aggravated for asylum seekers during pregnancy and early motherhood.

A complex array of social determinants of health at macro, meso and micro level potentially intensify pregnant women’s and new mothers’ vulnerability and influence their health status whilst seeking asylum. The World Health Organizations’ (WHO) *Conceptual Framework for Action on the Social Determinants of Health* [13] explains and conceptualises how social determinants influence health. Social determinants of health are factors linked to economic and social conditions that affect the health status of groups and individuals. On a macro level, nation’s socioeconomic policies affect the individuals’ health indirectly, for example via social policies on housing. On a meso level, socioeconomic status and social class indirectly affect health through a person’s purchasing power (or lack of) to access health services. On a micro level, material and psychosocial factors affect an individuals’ health, for example via distress disturbing an individuals’ sleep. Psychosocial stressors can arise on the macro, meso and micro level of the social determinants of health. This comprehensive WHO framework outlines social determinants of health which are also relevant when considering health-related needs of pregnant asylum seekers and new mothers.

Study results have highlighted the impact of social determinants of health. Focusing on housing as a social determinant of health is crucial as asylum seekers are mostly living in state-provided accommodation within the first weeks and months after arrival in receiving countries. Housing has been shown to play a decisive role for the mental health of asylum seekers and refugees residing in Australia and other resettlement countries [14]. Poor housing was identified as negatively affecting refugees’ and asylum seekers’ health while improving quality and security of the housing could ameliorate their health outcomes [14]. In Germany, a study on female refugees showed that women considered institutional living with overcrowded rooms and limited privacy with no place to retreat as burdening [15]. Housing, as one social determinant of health on the micro level, influences asylum seekers’ and refugees’ physical and mental health.

In Germany, asylum seekers are initially allocated at a state-provided reception centre. Due to a policy of compulsory dispersion, asylum seekers have limited influence over their geographic placement during the process of allocation [16] nor over the duration of their stay. The asylum seeking process may take six months [17] or longer [18]. Similar to other European Union (EU) countries, Germany applies policies of direct provision accommodation [19], which means its reception centres provide housing, meals and a minimal monthly allowance in cash to cover personal needs [20]. This housing is an institutional type of lodging with basic living conditions, catering (residents are restricted from cooking themselves), entrance controls and travel restrictions [16]. A condition of being accommodated in a reception centre is that the asylum seeker has to stay in the respective municipality or district [21]. In other EU countries, this direct provision accommodation comprising institutional living and institutional food has generated a debate that it infringes on asylum seekers’ rights to an acceptable standard of living, especially concerning the right to satisfactory housing and the corresponding rights to food and health [22]. Perceived psychological security and well-being and psychological and social stressor reduction in housing play a significant role in psychosocial health [23]. In addition, in Germany asylum seekers are transferred from reception centres to accommodation centres, shared flats with kitchens for self-catering and communal sanitary facilities. Transfers take place at any time after the official hearing, a personal interview conducted with each asylum seeker about the individual reasons for flight [24]. The interview is realised within two days after the formal application for asylum [18]. For pregnant women and those in early motherhood, each transfer adversely affects the continuity of their maternity care [6] and the maintenance of social contacts.

Recent systematic reviews focused on perinatal mental health in migrant populations and concluded that investigating in women’s mental health needs to be a priority [11, 12]. Furthermore, in Germany evidence on health and health-related needs of the particular group of asylum seekers that are pregnant or new mothers within the last 25 years remains limited [25]. The aim of the study was to investigate asylum seeking women’s experiences and perceived needs during pregnancy and early motherhood whilst living in state-provided accommodation in one federal state in Southern Germany, with a particular focus on psychosocial factors.

## Methods

### Design

This was a qualitative study with the objective of gaining in-depth understanding of the experiences and perceived needs of asylum seeking women during pregnancy and early motherhood. An exploratory case study [26] was selected as the research methodology. A particular strength of case study research is the flexibility it offers when investigating complex social phenomena, especially newly emerging phenomena or “cases”, while taking into account their real-life context. One of the most widely recognized definitions of case study research comes from Yin, in his classic text *Case study research*: *design and methods*. *1st edition* from 1984 and revised in 2014 [26]:

A case study is an empirical inquiry that

- investigates a contemporary phenomenon (the “case”) in depth and within its real-world context, especially when
- the boundaries between phenomenon and context may not be clearly evident (p.16).

Furthermore, data collection in case study research is typically associated with field work, which enables researchers to get “up close to the case being studied” [25], p. 24. This is a further reason why this methodology was regarded as appropriate. The “case” i.e. the complex social phenomena under investigation in this study was defined as: *the experiences and perceived needs of asylum seeking women during pregnancy and early motherhood* which was researched within the defined *context*: *state-provided accommodation including reception centres and accommodation centres for asylum seekers in Southern Germany*.

### Setting

The study was conducted in a federal state in Southern Germany that, according to administrative dispersal quota, received more than 10% of the total primary applications for asylum in Germany in 2016 [1]. Whilst an asylum seeking request is being processed, asylum seekers live at a state reception centre in Germany [27]. After having had their official hearing, which is relevant for the further course of the asylum seeking process, transfers to accommodation centres are organized by state authorities. In this study, participants lived in two out of ten reception centres spread across a federal state in Southern Germany, with an area of 35 000 km^2^ and an overall population of 10 million inhabitants, (comparable with Belgium or Switzerland) at the time of the first interview.

Reception Centre A consisted of ten housing units hosting over one thousand people. We interviewed women living in five of the ten housing units (Reception Centre A-1 to Reception Centre A-5). One housing unit (Reception Centre A-4 and later replaced by Reception Centre A-5) was reserved for vulnerable groups such as pregnant women from the late prenatal period (36^th^ week of gestation) or throughout the pregnancy in case of a high-risk pregnancy until the end of their postnatal period. Vulnerable groups also included disabled persons, both male and female. Reception Centre B housed more than one thousand people and offered a separate accommodation for single women either with or without children. Reception centres were organized as shared housing units, with generally six bunk beds per room. Reception Centre A and Reception Centre B both provided midwifery care during pregnancy and early motherhood.

### Participant recruitment

At Reception Centre A and B, the core research team established a working relationship with the leaders of the team of midwives before the beginning of the study. In particular, a “kick-off meeting” provided room for questions and answers related to the proposed research. In order to recruit study participants, data collectors from the research team were able to be present once per week during midwifery consultations at Reception Centre A and Reception Centre B from March to May 2016 and from May to July 2017. Through the attending midwife, they were provided with the opportunity to establish contact to potential study participants and invite them to consider being involved in the study. In discussions with potential participants, data collectors described their professional background and provided information about the study. An information sheet available in Arabic, English, Farsi, French, German, Kurdish and Serbian was distributed to potential participants. Potential participants were informed about study aims, data protection, use of data and potential benefits or risks linked with study participation. In total, 32 women received invitations and nine women gave their informed consent for study participation.

### Sampling strategy

The aim was to recruit 12 to 15 study participants for this exploratory case study, in which a purposive sampling strategy was applied [28]. In order to maximize diversity, SCG and ER purposively selected women based on “maximum variation” with respect to language and nationality [29]. Only asylum seekers who were in the first or second pregnancy trimester were included in order to create the potential for a second interview during pregnancy after a minimum of four weeks of time between interviews. The intention was to allow for women’s experiences to be explored over time during the asylum seeking process.

### Data collection

#### Field access

For the purpose of this study, access to asylum seekers living in state-provided direct provision accommodation was required. The research team applied to local authorities such as the regional councils and local health authorities for this purpose and access was granted duly.

#### Researcher characteristics

The core team was made up of two researchers: KB and SCG, supported by a Masters student (ER). KB is a male medical doctor with additional qualifications in public health and social epidemiology. SCG is a female medical doctor with additional qualifications in international health and has worked and lived in South East Asia and Sub-Saharan Africa. ER is a female studying Global Health at Masters level and has worked and lived in South East Asia. Time spent in South East Asian and Sub-Saharan African cultures enabled SCG and ER to bring their awareness of the diverse cultures in these regions to the research work. All three researchers have previously conducted research studies using qualitative approaches.

#### Interviews

Data was collected using individual semi-structured interviews. These were conducted using an interview guide and were digitally recorded. Topics in the interview guide for the three follow-up interviews were as follows: General well-being and personal situation; physical and mental health; social well-being; pregnancy; medical care in pregnancy; living environment; behavior and independency; personal characteristics; additional themes raised by the interviewee. During the second and third interview these topics were expanded to include the following question: What happened since the last interview? During the third interview further topics were: Delivery and hospital stay. Qualitative interviews were performed without any underlying framework. The three detailed interview guides are attached in a supplementary file. The two female interviewers started and ended each interview with open-ended questions allowing participants to raise and elaborate on topics that were relevant for them. Otherwise, interviews were conducted following the semi-structured interview guide. Continuity of interviewers and interpreters during follow-up interviews allowed for trust, acceptance and a sense of familiarity to be established. Eight women accepted digital voice recording. One study participant declined digital recording. In that case field notes and an interview summary were written after each interview.

Interview times and locations were arranged to suit the participants. The interview schedule is displayed in Table 1. Interviews were performed at the housing units in rooms that were selected in arrangement with interviewees and that allowed for privacy. Prior to starting each interview, participants were asked if they felt comfortable in the setting. We sought to perform three interviews with each asylum seeker: two interviews during pregnancy and one during early motherhood. A first interview was performed with nine asylum seeking women during pregnancy. Seven out of nine women lived at Reception Centre A and two women lived at Reception Centre B during the first interview. Interviews were conducted both at reception centres (Reception Centre A-1 to Reception Centre A-5 and Reception Centre B) and after women’s referrals at three different accommodation centres. Women in this study were not generally accommodated separately from men. At Reception Centre A, one housing unit (Reception Centre A-4 which was later replaced by Reception Centre A-5) was reserved for vulnerable groups including pregnant women from the late prenatal period (36^th^ week of gestation) or throughout the pregnancy in case of a high-risk pregnancy. Women stayed there until the end of their postnatal period if no transfer occurred in between. Prior to the 36^th^ week of gestation, pregnant women were accommodated in standard accommodation units that were shared with men. As vulnerable groups included disabled persons, the accommodation for vulnerable groups was not merely occupied by females, but also housed male asylum seekers. Reception Centre B offered a separate accommodation for single women either with or without children. Males were not allowed to access this accommodation. Families including father, mother and child/children were accommodated in standard housing units.

**Table 1:** Interview schedule.

**Study Participant**	T 1*	**Housing Unit**	T 2*	**Housing Unit**	T 3*	**Housing Unit**
SP1	X	RC_A_1	X	AC	X	AC
SP2	X	RC_A_1	X	AC	X	AC
SP3	X	RC_A_2	X	AC	X	AC
SP4	X	RC_A_3	-	-	X	RC_A_3
SP5	X	RC_A_3	X	RC_A_3	X	RC_A_3
SP6	X	RC_B	-	-	X	RC_B
SP7	X	RC_B	-	-	X	RC_B
SP8	X	RC_A_4	-	-	-	-
SP9	x	RC_A_5	-	-	X	RC_A_5

AC–accommodation centre; RC–reception centre; SP–study participant; T 1*—Interview 1 (prenatal); T 2*—Interview 2 (prenatal); T 3*—Interview 3 (postnatal)

Participants were asked if they were willing to provide a mobile phone number in order to follow-up on them for a possible second and third interview. All nine women agreed to be contacted for future interviews. Due to the advanced stage of pregnancy in five women, a second and final interview was conducted after delivery. One study participant dropped out from the study as she could not be reached by telephone after the first interview. In total, eight women were interviewed after delivery.

Data saturation was considered achieved as concurrent data analysis did not elicit new themes from the follow-up interviews.

#### Interview languages

The interview languages were Albanian, English, Kurmanji and Macedonian. For interviews which were not conducted in English, female professional interpreters assisted via telephone. When interviews were conducted with an interpreter, the same female interpreter did interpret all three interviews which assured the continuity of the interpreter for each woman throughout the data collection period.

### Data analysis

Digital recordings of interviews were transcribed verbatim using the transcription software f4 version 5 [30] and the interview transcriptions formed the basis for qualitative content analysis, which was supported by the qualitative data analysis software MAXQDA version 12 [31]. Data analysis was performed inductively, according to themes that women considered of greatest importance to them. At the stage of data analysis, we strove for embedding the findings in an existing theoretical framework. Therefore, themes that emerged during interviews were matched with a suitable framework for analysis. Due to the match between reported experiences of women and the WHO’s *Conceptual Framework for Action on the Social Determinants of Health* we considered that framework to be suitable. Data analysis was performed with an inductive approach to thematic analysis. According to Braun and Clarke we read and re-read the data for any themes related to relevant themes, generated initial codes, searched for themes, reviewed and refined themes, defined and named them in order to finally produce a report [32]. We performed inductive coding in an iterative process. Inductive coding included the following three steps. Firstly, we sought for patterns via open coding of the data, secondly, we established thematic categories and, finally, we determined core themes. In the initial step, we analysed transcripts via open coding of emerging and recurrent themes. In a second step, we organised these themes via identifying relations with each other and lastly, we performed selective coding to structure the data in core categories [33]. At each stage, we performed consensus discussions, related to content analysis within the team. Data collection and analysis were performed in a helical way, so that results from the ongoing data analysis could feed back into the following data collection strategy [34]. The interview guide was as a consequence modified and adapted throughout the study. After completing data analysis based on women’s narratives, significant parallels were identified to categories in the WHO Conceptual Framework for Action on the Social Determinants of Health [13]. This was then used as a theoretical framework for presenting results to the research community.

#### Techniques to enhance trustworthiness

To maximise trustworthiness of the data we applied the following criteria according to Flick et al. [35]: (a) communicative validation, (b) triangulation, (c) validation of the interview situation and (d) authenticity and trustworthiness:

In the course of the interviews, we frequently paraphrased and summarized the given information for clarification (*communicative validation* (a)). Via *investigator triangulation* (b) we sought to reduce “selective perceptions and blind interpretive bias” [36]. Data analysis and coding were performed separately by two persons of the research team. In addition, consensus discussions were supported by a third party. We strove for *source triangulation* (b) by recruiting interviewees at different locations and conducting our research at different points in time [37]. To *validate the interview situation* (c) the same team members and female interpreters conducted follow-up interviews with participants. Interviewers sought to establish an atmosphere of trust and openness during interview discussions. They paid attention to facial expressions and body language and responded to expressed needs of study participants in a caring manner. To foster *authenticity and trustworthiness* (d), the interviewers showed interest and respect in participants’ culturally diverging practices and attitudes during pregnancy and early motherhood. As mentioned above, interview locations (i.e. rooms) were selected to create an environment in which participants felt comfortable. Furthermore, debriefing conversations and team discussions about findings allowed for a rigorous reflection of content and situations and promoted our self-awareness throughout the research process. Our field notes of every interview contributed to a systematic audit trail [38]. Finally, in the preparation of results for publication, the consolidated criteria for reporting qualitative studies (COREQ) [39] and the standards for reporting qualitative research (SRQR) [34] were used.

### Ethics approval and consent to participate

Ethical approval for this study was obtained by the Ethical Committee of the Medical Faculty of Heidelberg University (S-688/2015). Prior to data collection we obtained asylum seekers’ informed written consent to participate in the study. We highlighted to participants that participation was voluntary and that neither participation nor non-participation would have an influence on health care provision or the course of the asylum seeking request. Participants received a small financial compensation for each interview.

## Results

We conducted a total of 21 semi-structured open-ended interviews with nine female asylum seekers during pregnancy and early motherhood between March 2016 and July 2017.

### Sample characteristics

Based on the maximum variation sampling, we interviewed a total of nine participants: four asylum seekers from West Africa, three from Southeast Europe, one from Western Asia and one from South Asia. Participants were between 22 and 37 years old (see Table 2).

**Table 2:** Participants’ biographic overview.

**Women’s ID code**	**Age (years)**	**Relationship status**	**Level of education**	**Previous occupation**	**Region of origin**
**SP1**	31	Single	secondary school	hairdresser	West Africa
**SP2**	35	Single	secondary school	student	West Africa
**SP3**	22	Married, living with husband and first child	primary school	homemaker	Western Asia
**SP4**	31	Married, living with husband	illiterate	homemaker	Southeast Europe
**SP5**	37	Single	secondary school	hairdresser	Southeast Europe
**SP6**	27	Married, living with husband	secondary school	hospitality	Southeast Europe
**SP7**	22	Single	primary school	hairdresser	West Africa
**SP8**	34	Married, living with husband and first child	university	homemaker	South Asia
**SP9**	36	Single	secondary school	cleaner	West Africa

ID–identification; SP–study participant

Participants had arrived in Germany one to six months prior to the first interview and were transferred up to four times. It was the first pregnancy for five out of nine women (see Table 3).

**Table 3:** Participants’ resettlement overview and obstetric history.

**Women’s ID code**	**Time living in Germany at 1**^**st**^ **interview (months)**	**Transfers until 3**^**rd**^ **interview (n)**	**Pregnancies (n)**	**Gestation at delivery (weeks)**	**Children (n)**	**Smoker (yes/no)**
**SP1**	2	2	1	39^th^	0	no
**SP2**	1	4	1	37^th^	0	no
**SP3**	1	2	2	38^th^	1	no
**SP4**	3	2	1	39^th^	0	yes
**SP5**	5	1	2	39^th^	1	yes
**SP6**	6	2	1	39^th^	0	no
**SP7**	3	2	1	38^th^	0	no
**SP8**	5	1	2	-	1	no
**SP9**	2	1	2	38^th^	1	no

ID–identification; SP–study participant

### Themes and categories

Based on the aim of the study, to investigate asylum seeking women’s experiences and perceived needs during pregnancy and early motherhood with a particular focus on psychosocial factors, we identified five themes in our content analysis that participants reported as affecting their health and well-being: a) Future uncertainties, b) stressful living circumstances, c) stressful relationship to child’s father, d) social support and e) coping styles. The established categories and associated sub-categories are displayed in Table 4.

**Table 4:** Themes, categories and sub-categories.

**Themes**	**Categories**	**Sub-categories**
**Future uncertainties**	• Lack of information • Upbringing of children	• Asylum seeking request outcome •
**Stressful living circumstances**	• Feelings of insecurity • Lack of self-determination • Lack of privacy	• Protection by security staff • Catering • Living Allowances • Noise • Sleep
**Stressful relationship to child’s father**		
**Social support**	• Social isolation • Professional support • Personal support	• Length of stay • Health professionals
**Coping styles**	• Religious faith • Hope • Acceptance	

#### Future uncertainties

Even though most participants reported happiness at being pregnant, they felt concerned about their unclear future. Uncertainty related to living conditions including both frequency and destination of referrals and the outcome of the asylum seeking process were major preoccupations. Participants felt they had minimal ability to influence their living situation:

“There is possibility that it (transfer) happens before the delivery and there is possibility it happens after the delivery… the woman downstairs, the social worker, she will …make it known to I don't know who, that if there is any space available that's there is a person waiting to be transferred yeah.” (SP2_I2)

A lack of information and transparency in the process of transfers was frequently described by participants:

“So for us, as far as I know in this moment there will be no, no transfer, but there is, there were women (transferred), but I don’t know to which place they were transferred- that remains unknown, that we don’t know either and we are waiting for it.” (SP4_I2)

Uncertainty was also reported by participants due to a lack of experience and understanding of norms and procedures in Germany. The language barrier exacerbated this issue:

“I don’t have an interpreter… I have thought about it but I did not make an effort to find an interpreter because I do not know the rules here.” (SP5_I2)

Stressful uncertain living conditions in the past were also reported by participants. One woman described unstable living conditions in her past and stated that she would like to overcome them for the sake of her child’s health and well-being:

“…because for many years you can see since 2002 I've been living from one country to another and I didn’t have a stable home so I don't want that for my child. I would like my child to live–me, my daughter, it's a girl, and her father—I would like us to live together.” (SP2_I1)

#### Stressful living circumstances

Participants frequently described feelings of anxiety in response to their current living conditions, which included exposure to verbal and even physical violence among residents:

“Yes I think that is true for everyone, so that you feel afraid and yes, that you do not really feel well… I have a difficult pregnancy and stay most of the time in the room and I am a little afraid of the neighbours because all the time they are shouting and they are hitting each other.” (SP6_I1)

Shared toilet facilities, located at the corridor, exacerbated women’s perceived difficulties whilst living at state-provided accommodations. Disturbances by other inhabitants of the reception centres sometimes seemed to develop to an extreme which, especially at night, required the intervention of security staff working at the accommodation units:

“I'm so happy that the security there were so caring… I just changed this (room) … it was just that I was kind of trauma because for many nights I never slept…” (SP2_I1)

A further source of stress for participants in their current living conditions was having little say over daily routines e.g. determining their own meal times:

“In (Reception Centre A) camp we eat… 7–9 o'clock breakfast time, 12–2 o'clock lunch time, evening 5–7. It's …difficult time, because 5–7 eat dinner. Sometimes we eat 5:30–6:30. After that I'm hungry because bread is not enough (when go to bed). Yeah. Very very hungry. Because that is not enough…” (SP8_I1)

In addition to the predetermined meal times, participants stated that they had very little influence over the type of food in their dietary intake, which was experienced as stressful, especially as being pregnant:

“No money so we have to just eats like, you see what I just eats, if I don't like it now that means I feel I'm not satisfied and have been eating bread, cheese, every … for the past two weeks. But sometime I don’t feel like eating it and I don't have any money with me so I feel like I'm not satisfied.” (SP2_I1)

Despite receiving a basic monthly living allowance, restricted financial circumstances created further difficulties for women:

“We don't get pay for now I don't know why… sometime I think about my cream, my lotion or because for now I don't have money to buy all these things I don't know when the money is coming. They say it will come but I don't know when …” (SP2_I1)

Women often referred to living conditions as “camps” and reported struggling with the limited privacy. The latter resulted from sharing their room with an unknown woman, several women or even a family, depending on the allocation of other asylum seekers. These living conditions contributed to the perceived stress in women’s crowded housing situations:

“It is better you are alone in the room so then you have your privacy.” (SP7_I1)

One participant described the challenge of being housed with three families from different cultural backgrounds in a single room:

„If there is a transfer it is possible that other persons are also accommodated in my room, so for example I had in the past three families that were accommodated, one from Syria, one from Albania and one from Serbia. Within that time it was very difficult for me.” (SP5_I1)

Constant background noise, especially from their neighbors at night, created problems in getting adequate rest for participants in reception centres:

“I would love to go somewhere else, so to have a transfer to somewhere else, somewhere where it is more quiet and where we (family) can be alone somehow. That I can sleep peacefully. At night sometimes the boys (other asylum seekers) are rioting, yes that happens.”(SP3_I1)

Interrupted sleep was reported as the norm by participants as one woman mentioned:

“Sometimes she (roommate) wash clothes ‘til three in the morning. Three a.m. she doesn't sleep and when I'm sleeping she is moving close to my head everywhere… My sleep …for now I do sleep also in the night when I don't have a lot themes in my mind. Yeah. So it depend on my mind, if I feel satisfied like okay I have enough for the day then I sleep good, but if I feel like something is missing I didn't get what I needed, not able to get it, then my sleep is half half it's kind of …Yeah is shorter.” (SP2_I1)

#### Stressful relationship to child’s father

In addition to stressors related to living circumstances, there were psychological stressors for participants regarding their relationship to the father of their child. These ranged from fleeing from the father due to the fear of his assaults to voluntarily living without the child’s father as he was considered an additional source of worries. One participant reported concerns for the safety of herself and her daughter:

“I feel especially insecure, when I leave the house (reception centre) to do some shopping, then I always have the feeling or the fear that he might be looking for me and he might find me. As for me this is not so bad but I am very much concerned about my little daughter… I only want to take care of my children and I hope that I will never meet him (husband).”(SP5_I1)

Many participants recognized the paternal “rights” of the father of their unborn child but expressed ambivalence towards being in a relationship with him:

“He (child’s father) is living here (Germany), but I don't want.. you know I don't … man is difficult… I want to see him, but I don´t want to stay with him…Because he is the father of my baby, I can’t deny that… (He) is not clean…men difficult, I don´t need problem, I don´t need stress.” (SP1_I1)

On the other hand, other participants communicated worries about being a single mother:

“I can’t feel well, my head is overloaded… I don’t know. The thing is, the reasons that I said and how my children will grow up, that means now two kids, not with two parents, growing up with a single mother that makes me worry.” (SP5_I1)

In each case of the four study participants who were married and applied for asylum with their husband (and in two cases their child), couples or families were accommodated jointly in one room.

#### Social support

According to participants’ opinions, building social contacts and friendships proved difficult because of the short length of stay in reception centres (three weeks to six months) and the frequency of transfers between accommodations (with up to four transfers between different cities). This complicated the establishment and continuity of social supports for these women. Some explicitly described their perceived isolation:

“No lots of human greet… .just greet “Hello hello”… I sit my own for lunch and breakfast …I sit alone.” (SP1_I1)

Establishing desired social support networks was also perceived as challenging by participants resulting from factors such as the multitude of nationalities and the language barriers:

“…because in (city) people from Afghanistan, Macedonia they don't speak English …only languages … Some people speak German …language. So very difficult to contact, yeah. Very little people have …can contact. It's very difficult.” (SP8_I1)

However, dealing with such psychosocial stressors was made easier when personal support was received. It was appreciated when people from the local community reached out with support:

“It’s a man, a friend, German. I do trust him, he is a very nice person, very nice to me. He is very nice. He told me if I need any help, I can call him.” (SP1_I3)

Professional support offered by social workers, psychologists and midwives also played a significant part in allaying fears and reducing psychological stressors. One woman reported accessing needed psychological care due to an observant social worker:

“Yes she (name social worker) saw that I was very stressed and very very sad. (Name social worker) when she asked me, she told me „we (social workers) have seen that you have completely changed“. (Name social worker) has called me into her room and told me “we (social workers) see that you do not smile at all and that you do not feel well. What is going on with you?” She asked me to talk about my problems, to say what my problems are.” (SP5_I2)

Another participant highlighted the positive influence on her mental state by talking to a psychologist:

“It helps me very much when she (psychologist) comes, she puts my mind at ease, calms me”

The professional care from midwives was also experienced as beneficial:

“Yeah. Because I think, they help lot—the midwife… They give advice and they check all my body—and I think it's good… And the advice how to control my urine infection—a lot of water and they checked my urine every week. And I ask because urine infection not good for pregnancy life. That's why I a little bit afraid. And it's normally—you drink a lot. No, no. Now it's okay.” (SP8_I1)

#### Coping styles

Participants shared their own ways of dealing with the stressful and challenging conditions of being an asylum seeker. Acceptance of the current circumstances was mentioned as one method of coping:

“We are eight people sleeping in the same room, but two people prefer to sleep in the sitting room, so… with eight people it's normal, sometime one want to go to the toilette or one is doing this. Yeah, so there is a bit distraction but you just have to … we just have to accept it and live with it.” (SP2_I2)

An additional coping style that helped several women to endure their situation was their religious faith:

“Here (reception centre) is not too comfortable for me… (I wish) that I just make my transfer. That is what I pray for; I need the transfer…I pray that they should make my transfer before (delivery)… I expect they take me to a living house not camp…” (SP1_I1)

All participants communicated the strong wish for an early transfer from the reception centre to an accommodation centre with the hope for better living conditions before the date of delivery. None of them knew the date or destination of their transfer neither the living conditions at the accommodation centre yet they hoped for an improvement compared to the living conditions at the reception centre.

Due to the experienced uncertainties and limited self-determination, several women expressed their hope for an improvement of their current situation in the future:

“I hope, after six months they give a start. I think, it's good, because a pregnant woman needs to be very difficult in camp life. I don't know, very confused. I don't know the transfer start I'm not sure, but my idea is pregnant woman will give a start because very very difficult in a camp—for pregnancy … (Change after transfer) We can cook—is the main thing.” (SP8_I1)

## Discussion

This exploratory case study provides in-depth insights into the experiences and perceived needs of asylum seeking women during pregnancy and early motherhood with a particular focus on psychosocial factors whilst living in one federal state in Southern Germany. Key findings from this study showed that these women perceived psychosocial factors based on social determinants of health as negatively affecting their health and well-being. Asylum seeking women in our study experienced a range of psychosocial stressors in daily life. They perceived uncertainties of the future due to a lack of information and transparency linked with the heteronomous accommodation and frequency and destination of referrals during the asylum seeking process and an unknown outcome of the asylum seeking request. This was aggravated by a limited understanding of norms and procedures in Germany. In addition, asylum seeking pregnant women and new mothers in our study experienced problems caused by stressful living circumstances with a perceived lack of privacy due to sharing rooms with unknown families or women. As an aggravating factor to the experienced lack of privacy, accommodation units with both male and female asylum seekers caused a feeling of anxiety in our interviewees, affecting women’s sleep negatively in the direct provision accommodation. Furthermore, participants experienced restrictions regarding financial circumstances, a lack of self-determination and little individual choice e.g. related dietary intake, which they felt deprived them from the possibility of satisfying their basic needs during pregnancy and early motherhood. Additionally, stressful relationships with the child’s father were experienced as an additional burden by our participants, yet raising the children as a single mother was also considered as worrisome. Moreover, a perceived social isolation was exacerbated by frequent transfers and language barriers affecting women’s psychosocial health negatively. In our study, participants highlighted the value of professional support to access care for their mental and physical health needs. To counterbalance their perceived psychosocial stressors, most study participants stated the importance of individual coping styles such as acceptance, religious faith and hope for the future.

Similarly to our findings, a recent report on female refugees in Germany showed that these women perceived their future as uncertain [40]. In particular, short-term residency permits and fear of deportation were experienced as demoralizing [40]. The United Kingdom’s National Health Service has also stated that a drawn-out asylum seeking process and the fear of negative decisions caused “daily anxiety and uncertainty about the future, fear of deportation and future safety” [41], which negatively affected asylum seeking women’s well-being. Nevertheless, there seems to be a trend towards “more restrictive asylum policies, including increased periods of mandatory detention, extended processing times, and the implementation of temporary (rather than permanent) visas for refugees” [42]. These immigration policies and the length of time taken for the decision to grant asylum are considered to negatively affected refugees’ mental health status [42]. A national report of female refugees in Germany also recommended that the asylum seeking process be accelerated to reduce the influence of post-migration stressors and the prevalence of psychological impairment [40]. In addition, results from a study on asylum seekers in the Netherlands showed that stress linked with the procedure of applying for asylum had the highest odds ratios for psychopathology causing authors to “appeal to governments to shorten the asylum procedures” [43]. Social determinants of health at the macro level, namely immigration policies, cause women to experience future uncertainties during the asylum seeking process and contribute to asylum seekers’ psychosocial stress.

In accordance with our findings, which revealed women’s perceived lack of information and transparency concerning the asylum seeking process, the 2017 report on female refugees in Germany highlighted that women who applied for asylum experienced the lack of information related to the asylum seeking process as problematic [40]. Women referred to in the report also desired measures such as improved transparency related to their application process [40]. Furthermore, it was found that women faced difficulties due to language barriers, which complicated the process of them gaining understanding of what government departments expected from them [40]. Similarly, a study on refugees in the United Kingdom reported that “difficulties with the immigration system, housing and social services” [44] were common and that refugees considered the “stress of adaptation and settlement” [44] as a negative influence on their mental health, potentially leading to depression. Language barriers and unfamiliar bureaucratic systems have been reported as factors negatively affecting on information transfer, which contributed to psychosocial stress.

In general, as stated in the WHO’s *Health Principles of Housing* [23], adequate accommodation should minimise people’s psychological and social stressors related to their housing environment and protect populations at special risk such as pregnant women. Prenatal anxiety has been found to be positively related to an elevated risk of pre-term birth and low birth weight [45]. This has implications for migrants and asylum seekers. Post-migration living difficulties have been linked with high rates of psychological distress in refugees resettling in Australia [46]. In particular, housing has been shown to play a crucial role for the mental health of asylum seekers and refugees living in Australia and other resettlement countries [14]. Poor housing was found to affect refugees’ and asylum seekers’ health negatively whereas improving quality and security of the housing could improve their health outcomes [14]. A study on female refugees in Germany found that women considered institutional living as stressful, with overcrowded rooms lacking privacy with no place to retreat [15]. A report from Pro Asyl, a German human rights organisation, has confirmed that privacy was largely lost for refugees in institutional living circumstances, exacerbated by the constricted shared facilities [16]. It has been reported by the EU Directorate-General for Internal Policies, in a case study on Germany, that prolonged asylum seeking procedures, marked by direct provision accommodation with a lack of activities for asylum seekers, may cause boredom in asylum seekers, which in combination with stress can trigger violence [47]. A study on refugees’ and asylum seekers’ mental health in Australia identified violence and threats in the resettlement environment as negative influences [48]. In summary, social determinants of health at the macro and micro level, namely immigration policies and material factors such as close living quarters, generate a range of challenges that leads to psychosocial stress in the institutional living circumstances of asylum seekers.

A further source of psychosocial stress identified in our study was linked with cultural diversity in an institutional living environment. This is also mentioned in recent findings by Schouler-Ocak and Kurmeyer, who reported that this resulted in tensions, discrimination, name-calling and other conflicts between residents in the direct provision accommodation [40]. In addition, they reported the problem of a lack of gender-based separation in direct provision accommodation facilities for female asylum seekers and described the frequent lack of separated sanitary facilities as stressful for many women [40]. This caused mothers in particular to worry about their daughters’ security, generating the strong wish for a private apartment or separated accommodation for women. This report also highlighted the need for a stronger focus on a specific gender-based strain related to women’s needs in these accommodations. In another study, female gender and post-migration stress were associated with post-traumatic stress disorder, depression and anxiety symptoms [49]. Diversity and a lack of gender-based separation in housing and the resulting tensions among asylum seekers in institutional living, representing social determinants of health at the micro level, namely psychosocial factors, further contribute to women’s perceived psychosocial stress.

Furthermore, our participants stated to have experienced limited self-determination (e.g. economic and social restrictions) in the institutional living circumstances. The EU Directorate-General for Internal Policies declared that inflexible rules and conditions add to the stressors in reception centres [47]. Refugees and asylum seekers in Australia mentioned financial restrictions as a central post-migration stressor [48]. Stressors in the resettlement environment in Sweden were similarly marked by social and economic strain, which have been attributed to symptoms of mental disorder in refugees and asylum seekers [50]. The stress-related influences of socioeconomic difficulties on refugees’ mental health outcomes were found to be significant [42]. The United Kingdom National Health Service have attempted to address this problem by offering an additional allowance for pregnant women or those with children under three years [41]. Social determinants of health at the macro level, such as the lack of self-determination connected to institutional living and institutional food as well as those at the meso level, namely socioeconomic restrictions, are all factors acting as psychosocial stressors for asylum seeking pregnant women and new mothers.

Moreover, further psychosocial stress can arise in pregnant asylum seeking women and new mothers that are single and juggling multiple responsibilities both for themselves and their children. This was illustrated by the five single mothers in our study. Similarly, it has been reported that being separated from their families was experienced as a significant psychosocial stressor for asylum seeking women [40]. In many cases, these women had full responsibility for children who fled with them. Researchers found that parents who recently immigrated have been at risk for “emotional health problems during the post-migration period” and that “parents have even higher odds of reporting emotional problems compared to non-parents, and lone parents are at the greatest risk” [51]. Single and unmarried women and those lacking emotional support within the partnership were at risk of perinatal mental health disorders [52]. It was determined that “lone parent respondents had odds of reporting emotional problems that were over twice as high in comparison to respondents that were non-divorced, non-parents [51]. A study on Southeast Asian refugees in Canada has revealed that “a stable relationship helped protect individual mental health during the process of temporal reintegration” [53]. Whereas a study on Pakistani immigrants in Norway showed that those being unmarried and having a poor partnership disposed of a significant risk of postpartum depression [54]. Stressful relationships, displaying the social determinants of health at the micro level, create further stressors for pregnant women and new mothers during the asylum seeking process.

As highlighted in our study, social support is of great importance for women seeking asylum. This has been also reported in a recent study stating that policies around dispersal and no choice accommodation hindered women’s opportunities for establishing social support networks [55]. It has been reported that Southeast Asian refugees who have come to Canada and have lacked “personal and social supports suffered an increased risk of depression 10 to 12 months after arrival” [53]. In addition, a lack of social support may lead to feelings of anxiousness [56]. Several studies have reported social isolation in refugees in the post-migration period and the challenging effects on their mental health [6, 46, 57, 58]. Furthermore, lack of contact with one’s own culture or a community offering support networks is considered as having an adverse effect on women’s well-being [41]. Loneliness has been identified as a post-migration stressor [48]. Muslim refugee women in Australia repeatedly mentioned “feelings of isolation and a lack of belonging” [59]. Frequently, a perceived language barrier increased the feeling of isolation adding a sense of “dependency on other people and the inability to mix with people of other language backgrounds” [59]. The post-migration and resettlement phase are marked by uncertainties and appropriate social support can offer an integral approach to health and can improve quality of life [60]. This is especially important for pregnant women, where a lack of social support is a relevant prenatal risk factor that has negative effects on pregnancy outcomes [61]. Mothers who received low social support during early pregnancy gave birth to babies who weighed up to nearly 200 g less at birth [61]. Inadequate social support and the impersonal living conditions of state-provided accommodation, social determinants of health at the micro and meso level, are a further cause of psychosocial stress for asylum seeking pregnant women and new mothers.

Social support offered by professionals, such as midwives, social workers and psychologists was one means that counterbalanced psychosocial stress for our study participants. These findings are supported by a study from the United Kingdom that concluded that maternity care teams and voluntary services were essential to ensure continuity of care and support whilst providing obstetric care for asylum seekers and refugees [6]. Moreover, a study on Bosnian refugee women has reported social support was a crucial resilience factor for women, which helped them adapt during resettlement [62]. The continuity of women’s maternity care [6] and social supports is negatively affected by each transfer between reception centres and to accommodation centres. As access to and continuity of health care and professional social support is helpful for both physical and mental health of pregnant women and new mothers in the post-migration phase, social determinants of health at the macro level, namely immigration policies regulating accommodation in reception centres and accommodation centres and timing of transfers should consider the relevance of continuity in health care and social supports.

The relevance of individual coping styles was a key finding in our study and has been recognised by other researchers. A study on Somali refugees and asylum seeking women reported women’s ability to move on and cope with the current situation as fundamental for their psychological well-being and religious faith was described as supportive [63]. Resilience strategies identified by other researchers also included faith and hope as sources of strength for refugees [64]. Pregnant women and new mothers seeking asylum have demonstrated a range of individual coping styles, a social determinant of health at the micro level, to counterbalance the psychosocial stressors experienced in their daily life.

As declared in a directive by the European Union parliament, European Union member states ought to take the specific situation of vulnerable persons including pregnant women and single parents with minor children into account and should consider their special reception needs and consequently offer specific support [65]. Therefore, implications for policy makers are that immigration policies should take psychosocial determinants of health into account to cover the health needs of vulnerable refugees and asylum seekers including pregnant women and new mothers. Policies should also consider the number and timing of transfers during pregnancy and early motherhood as they jeopardise the continuity of care and social support. Implications for research groups are that further research is necessary to establish both quantitative and qualitative evidence on these women’s health-related needs, access to, provision of and equity of care. Additionally, future studies including a human rights and gender perspective may further add to improving health and well-being of this vulnerable group. Such future research findings would help to develop awareness of these women’s special risks and health needs and contribute to a sound evidence base for decision-makers.

A strength of this qualitative study is its in-depth focus on the impact of social determinants of health on asylum seeking women during pregnancy and early motherhood to shed light on both women’s lived experiences and associated consequences on their health and well-being whilst living in a reception centre or accommodation centre. The WHO’s *Conceptual Framework for Action on the Social Determinants of Health* [13] that supported data analysis, if also drawn upon by other researchers, can assist international comparisons and research development in this field. Although this is a qualitative study on asylum seeking women in one federal state in Southern Germany and results are not generalisable to a wider population, it is possible that there are commonalities for asylum seeking women during pregnancy and early motherhood, which may be of use to service providers and researchers in other regions. The findings of this exploratory case study research must be viewed under the specific quality criteria applying to qualitative research, which were applied in this study to maximise trustworthiness and to ensure the quality of our findings.

Nevertheless, some limitations must be considered when interpreting these findings. Restricted finances have been shown in the literature to be a source of psychosocial stress for asylum seekers [48]. This also proved to be the case in our study. Participants received 20 Euros compensation per interview, which was a financial incentive to participate. This may have influenced the willingness to participate in the study. Furthermore, using professional female interpreter services enabled us to bridge language barriers, yet there is a risk of loss of information due to interpretation and it is unknown in which way these interpreter services may have influenced participants’ statements which formed the basis of analysis. Due to the fact that there were nine study participants, results need to be interpreted with caution. As results emerged out of a specific context it should be taken into account that results from a similar study undertaken elsewhere could vary considerably. Nevertheless, this small sample size enabled us to collect in-depth data. Despite these limitations, our unique insights contribute to the evidence base available for determining health-related policies and to inform health care providers about this vulnerable group. Further studies investigating health-related consequences caused by the migrant trajectory affecting social determinants of health and using both qualitative and quantitative approaches are needed.

### Conclusions

This exploratory case study provides in-depth insights into the experiences and perceived needs of the highly vulnerable group of pregnant asylum seekers and those in early motherhood living in state-provided accommodation in one federal state in Germany. Key results identified psychosocial factors, such as future uncertainties, stressful living circumstances and stressful relationships, as social determinants of health that based on study participants’ statements affected their health and well-being negatively. Adequate social support and individual coping styles increased resilience and counterbalanced psychosocial stressors for pregnant asylum seeking women and new mothers in daily life during the asylum seeking process.

## Abbreviations

AC: Accommodation centre
CSDH: Commission on Social Determinants of Health
EU: European Union
ID: Identification
RC: Reception centre
SDH: Social determinants of health
SP: Study participant
WHO: World Health Organization
